# Neuroendocrine Tumor of the Ureter: A Zebra Among Horses

**DOI:** 10.1089/cren.2016.0103

**Published:** 2016-11-01

**Authors:** Akshay Sood, Sean R. Williamson, David A. Leavitt

**Affiliations:** ^1^ Vattikuti Urology Institute, Henry Ford Health System, Detroit, Michigan.; ^2^ Department of Pathology, Henry Ford Health System, Detroit, Michigan.

**Keywords:** neuroendocrine tumor, small cell carcinoma, ureter, upper urinary tract

## Abstract

Primary neuroendocrine tumors of the upper urinary tract are extremely rare. We report a case of *de novo* small cell carcinoma of the ureter that presented masquerading as a distal ureteral stone. A 55-year-old lady presented to our clinic with 1 month history of right lower back pain and hematuria. Her history was notable for stage 1B mixed clear cell-endometroid cancer of the uterus status post radical abdominal hysterectomy with adjuvant radiotherapy, 7 years before the current episode. The patient had no evidence of recurrence. Initial noncontrast imaging suggested a 2.5 mm calculus in the distal right ureter and hydronephrosis; however, ureteroscopy revealed a large fleshy mass at the location. Histopathologic evaluation demonstrated the lesion to be primary small cell carcinoma of the ureter, without evidence of it being a derivative of the prior gynecologic malignancy. Metastatic work-up revealed high burden retroperitoneal adenopathy. The patient was started on Cisplatin-based neoadjuvant chemotherapy with plan for nephroureterectomy in the future. At 3 months follow-up, the patient was doing well with significant shrinkage of retroperitoneal adenopathy and no evidence of disease progression.


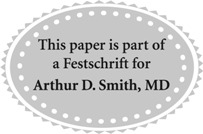


## Introduction

Neuroendocrine tumors arise from neuroendocrine cells.^1^ Common examples include small cell carcinoma of the lung, carcinoid tumor of the small intestine, pheochromocytoma of the adrenal gland, and medullary carcinoma of the thyroid. Primary neuroendocrine tumors of the urinary system are uncommon, accounting for less than 0.05% of all urinary tract malignancies.^2,3^ Primary ureteral neuroendocrine tumors are furthermore rare, with less than 30 cases having been reported since the initial description of these tumors in the mid-1980s.^3^ Herein, we report a case of small cell carcinoma of the ureter and discuss the clinical presentation, etiology, prognosis, and management of these uncommon tumors.

## Case Report

A 55-year-old lady presented to our clinic, referred from the emergency department, with 4 weeks duration of intermittent right lower back pain, fatigue, and associated nausea. There was no associated dysuria, urinary urgency or frequency, or fevers; however, she reported possible hematuria in the guise of “orange” urine. She denied abnormal vaginal discharge, constipation, or recent trauma. She denied a history of prior urolithiases or recent urinary tract infections (UTIs). She had never smoked tobacco. She denied recent weight loss.

**FIG. 1.**
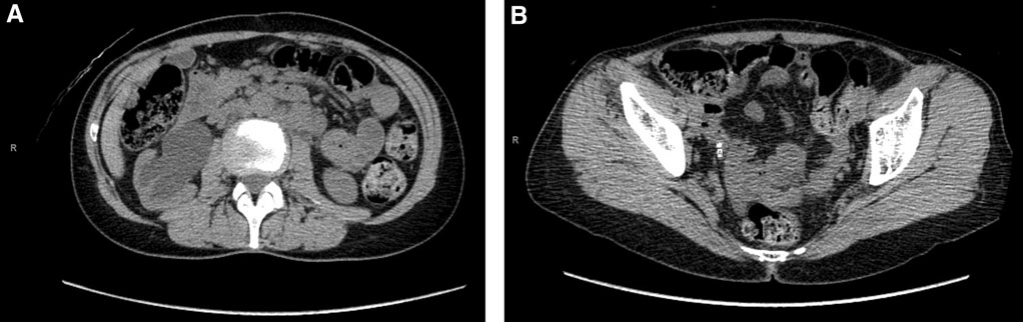
Noncontrast CT scan at initial presentation, demonstrating **(A)** severe hydroureteronephrosis and **(B)** a 2.5 mm distal ureteral stone.

Her medical history was notable for stage 1B mixed clear cell-endometroid cancer of the uterus status post total abdominal hysterectomy, bilateral salpingo-oophorectomy, and bilateral pelvic/para-aortic lymph node dissection (0 of 22 lymph nodes positive) with adjuvant radiotherapy, 7 years before the current episode, with no evidence of recurrence. An abdominal and pelvis CT scan revealed severe right-sided hydroureteronephrosis, marked renal cortical thinning, and a 2.5 mm distal ureteral calculus (Fig. 1A, B). The urinary bladder and the left renal unit appeared normal, and no lymphadenopathy was recognized. Urinalysis was negative for infection; however, four red blood cells per high-power field were appreciated. Creatinine was stable at 0.90 mg/dL. White and red blood cell counts were stable at 3700 cells per microliter and 13.2 gm per deciliter, respectively.

**FIG. 2.**
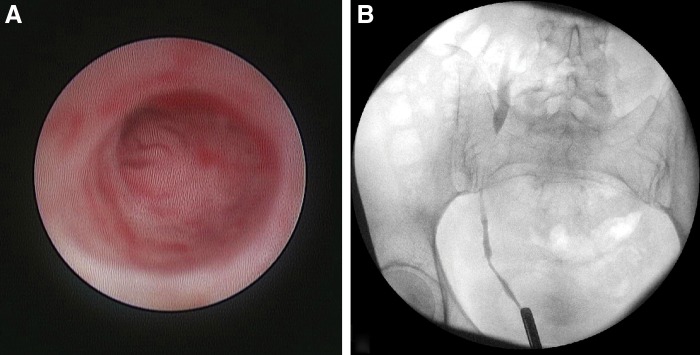
Intraoperative ureteroscopy revealing a mass in the distal ureter **(A)** and retrograde pyelogram showing a filling defect and extent of ureteral tumor involvement **(B)**.

To better assess right renal function, the patient underwent an MAG-3 renogram that showed severely decreased right renal function with a split renal function of ∼20%, and an excretion half-life of >30 minutes. Subsequently, the patient was taken to the operating room for a right-sided diagnostic ureteroscopy and possible stone lithotripsy. Intraoperatively, however, no stones were observed, rather, a fleshy mass was observed in the distal ureter with proximal hydronephrosis (Fig. 2A, B). Biopsies were obtained from the lesion and a plastic, double pigtail ureteral stent was left in place.

**FIG. 3.**
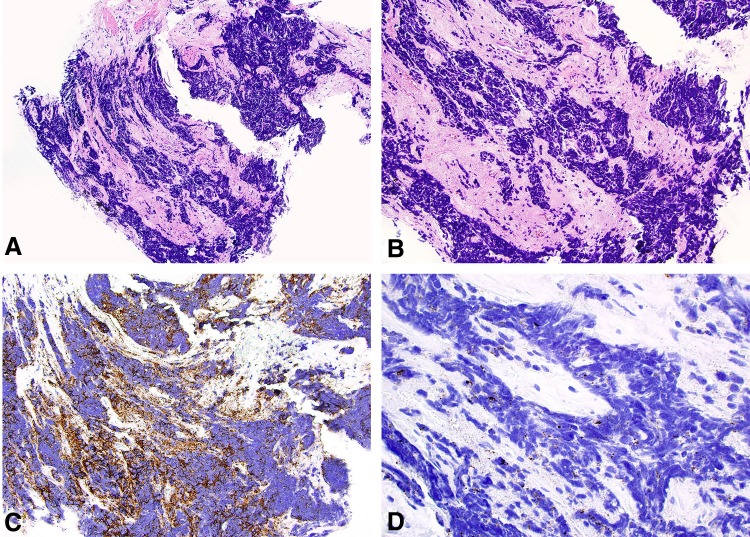
**(A)** Low magnification of the ureteral biopsy showing small blue cells with substantial crush artifact infiltrating fibrous tissue, **(B)** at higher magnification, cytoplasm is scant and nuclear molding is present, **(C)** immunohistochemical staining revealed diffuse positivity for synaptophysin, and **(D)** only minimal dot-like positivity for EMA, consistent with small cell carcinoma. EMA, epithelial membrane antigen.

**FIG. 4.**
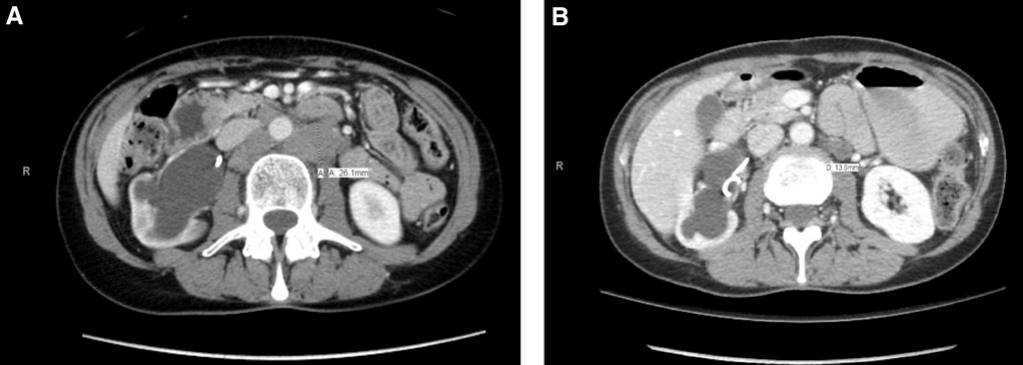
Metastatic work-up after the diagnosis of small cell carcinoma with contrast-enhanced CT of the abdomen–pelvis showing extensive retroperitoneal adenopathy **(A)**, response to chemotherapy demonstrating ∼50% reduction in the lymph nodal burden **(B)**.

The histopathology demonstrated small round blue cells with hyperchromatic nuclei, most consistent with pure small cell carcinoma of the ureter. The immunohistochemical staining revealed positivity for synaptophysin that supported the neuroendocrine phenotype; staining for urothelial carcinoma (GATA 3 and p63), gynecologic cancer (PAX8), lymphoma (PAX5 and LCA), or sarcoma (Myogenin, CD31 and Desmin) was negative (Fig. 3). Given the findings, a full work-up for metastatic disease was initiated and included: (1) contrast-enhanced CT of the abdomen–pelvis revealed bulky retroperitoneal lymphadenopathy involving retrocaval/aortocaval/para-aortic nodes extending from the level of the ureteral lesion (L5–S1) to the right common iliac vessel bifurcation (Fig. 4A), (2) contrast-enhanced CT of the head and chest was negative for metastatic disease, and (3) the Tc-99 bone scan was negative for bony involvement. The final clinic stage was cT3N2M0. The patient was started on Cisplatin–Etoposide neoadjuvant chemotherapy that the patient tolerated well except for minor hearing loss, with a plan for nephroureterectomy pending favorable clinical response. At 3 months of follow-up, she was doing well with lymphadenopathy reduction of 50% (Fig. 4B) and no evidence of disease progression.

## Discussion

As with urothelial carcinomas of the ureter, patients with neuroendocrine tumors of the ureter often present with flank, back, or abdominal pain, weight loss/fatigue, hematuria, dysuria, or recurrent UTIs. Overall, pain and hematuria are the most common initial symptoms.^3,4^ However, unlike urothelial carcinomas, locally advanced or nodal disease at presentation is common, with an estimated 20%–45% of patients harboring advanced disease at the time of diagnosis^5,6^—these findings are in line with the aggressive behavior demonstrated by neuroendocrine tumors arising from other sites.^7^

In contrast to other common neuroendocrine tumors, however, the primary neuroendocrine tumors of the urinary tract are rarely associated with paraneoplastic syndromes.^8^ Most primary neuroendocrine tumors of the ureter occur in the sixth to seventh decade of life, and there does not appear to be a gender predilection.^3,4^ Our patient thus demonstrated the classically reported clinical features; however, the age was premature. Although the symptoms that our patient presented with may be classic in patients with neuroendocrine tumors of the upper urinary tract, benign diseases by far are a more common cause of such symptomatology.

Remarkable about the present case is the fact that final pathology revealed *de novo* small cell carcinoma of the ureter, a very rare entity, whereas the patient had myriad more probable causes of unilateral ureteral obstruction, including obstruction secondary to ureteral stricture disease in the setting of prior radiotherapy and gynecologic surgery, stone disease, and recurrence of prior uterine disease causing intrinsic or extrinsic ureteral compression. This highlights the importance of a high index of suspicion in these cases. The only reported risk factor for this tumor is smoking; however, these data come from small cell carcinoma of the bladder,^9^ and for neuroendocrine tumors of the upper urinary tract, this association is, at best, tenuous.^10^ Our patient was a nonsmoker.

Pathogenesis of these tumors also remains debated, as it is thought that ureters normally do not harbor cells of the neuroendocrine system. Several hypotheses have been proposed to explain the occurrence of these tumors in the ureter; these include (1) neuroendocrine metaplasia of urothelial carcinomatous lesion, (2) seeding from normal neuroendocrine cells present in the urinary tract that later turn malignant, (3) entrapment of neural crest-derived cells in the ureter during embryogenesis that later turn malignant, and (4) from undifferentiated stem cells that differentiate toward a neuroendocrine lineage secondary to Notch1 mutations.^11,12^ The rarity of the disease has limited any meaningful conclusions, and these hypotheses have remained just that.

With respect to treatment, there is a lack of consensus regarding the ideal management of ureteral neuroendocrine tumors; however, contemporary case reports seem to suggest that a favorable strategy includes utilization of Cisplatin-based neoadjuvant chemotherapy followed by nephroureterectomy. These case reports utilizing Cisplatin-based neoadjuvant chemotherapy followed by surgery have reported disease-free survival times of 24 to 38 months,^13,14^ whereas a prior study of 39 patients with small cell carcinoma of the upper urinary tract reported an overall survival time of only 15 months in patients managed with surgery alone or surgery with adjuvant chemotherapy.^5^ Although validation in larger cohorts is needed, taken together these findings suggest that early institution of chemotherapy may be pivotal in improving survival in these patients. Furthermore, it may help identify patients responding to chemotherapy and, therefore, possibly more likely to tolerate and benefit from surgical extirpation compared with those who exhibit disease progression despite chemotherapy, and thus may not benefit from surgery. The choice of chemotherapy regimen seems crucial as well. Lastly, the follow-up protocols for these patients are not standardized, but a pragmatic one should consist of routine clinic visits, imaging and cystoscopic evaluation approximately every 3 months, given disease recurrence in upward of 50% of the patients, despite treatment.^5^ Our patient was started on Cisplatin–Etoposide regimen and a nephroureterectomy is planned if she continues to respond well. At 3 months follow-up, the patient was doing well on the chemotherapy regimen with reduction of lymph node burden on imaging and without evidence of disease progression.

## Conclusion

Neuroendocrine tumors of the upper urinary tract remain uncommon and present a diagnostic challenge. Risk factors and pathogenesis are poorly understood because of the rarity of the disease. Clinical symptoms and radiographic findings are nonspecific and are those related to ureteral obstruction; however, a short duration of symptoms with evidence of nodal disease on imaging may hint toward a neuroendocrine phenotype. Although disease recurrence and dismal survival have been the norm in the past for patients treated with surgery alone or with surgery and adjuvant chemotherapy, recent case reports utilizing Cisplatin-based neoadjuvant chemotherapy with nephroureterectomy have demonstrated extended disease-free survival and provide some hope for patients with this aggressive disease.

## Abbreviations Used

MAG-3: mercaptoacetyltriglycine
GATA: globin transcription factor
PAX: paired box
LCA: leukocyte common antigen
CD: cluster of differentiation
UTI: urinary tract infection
CT: computed tomography
